# Atypical Hemolytic Uremic Syndrome in a Patient With Metastatic Peritoneal Serous Carcinoma: A Case Report

**DOI:** 10.7759/cureus.22624

**Published:** 2022-02-26

**Authors:** Zachary H Daily, Syed Shahrukh Rizvi, Rafael Baez-Bonilla

**Affiliations:** 1 Nephrology, Lake Erie College of Osteopathic Medicine, Bradenton, USA; 2 Nephrology, Holy Cross Hospital, Fort Lauderdale, USA

**Keywords:** eculizumab, microangiopathic hemolytic anemia, peritoneal serous carcinoma, kidney failure, thrombotic microangiopathy, atypical hemolytic syndrome

## Abstract

Atypical hemolytic uremic syndrome (HUS) is a part of a large category of diseases known as thrombotic microangiopathies that present with hemolytic anemia, thrombocytopenia, and target organ damage mostly characterized by acute kidney injury. It is a rare and challenging diagnosis due to the complex pathophysiology underlying the disease and its overlap with other conditions. We report the case of atypical hemolytic uremic syndrome in a 61-year-old female with a history of metastatic peritoneal serous carcinoma of the ovary presenting with anemia and acute renal failure.

## Introduction

The diagnosis of Hemolytic Uremic Syndrome refers to a presentation of microangiopathic hemolytic anemia (MAHA) combined with thrombocytopenia and acute kidney injury [1]. It is a subclassification of thrombotic microangiopathies (TMA), a broad group of abnormalities in the vessel walls of arterioles and capillaries. Hemolytic uremic syndrome (HUS) may be classified as typical or atypical. Typical HUS is caused by Shiga-toxin-producing *Escherichia coli*. Atypical HUS encompasses all other causes of the syndrome, including inherited gene mutations of complement, autoimmune antibodies to complement factors, drug toxicity, and rare occurrences in pregnancy [2].

Typical HUS has an incidence of 2.1 cases per 100,000 persons per year [3]. It is highest in children younger than five and lowest in adults 50 to 59 years of age. On the other hand, atypical HUS accounts for only 5%-10% of all HUS cases. It can be sporadic or familial. However, its incidence is only two cases per 1,000,000 persons per year [3].

The pathogenesis of complement-mediated HUS involves the overactivation of the alternative pathway of complement after a triggering insult such as an infection in genetically predisposed individuals. Complement overactivation damages the renal endothelium and activates the coagulation cascade. The most common variant is due to the inactivation of complement factor H, which binds C3b and decays C3 convertase [4-6]. The clinical presentation involves a triad of hemolytic anemia, thrombocytopenia, and acute kidney injury. Further lab testing for specific markers, including ADAMTS13 (a disintegrin and metalloproteinase with a thrombospondin type 1 motif, member 13), coagulation studies, and genetic testing, can help to establish a diagnosis [7]. Atypical HUS has been linked to several distinct genes involved in the complement pathway, including C3, CD46 (MCP), CFB, CFH, CFHR1, CFHR3, CFHR4, CFHR5, CFI, DGKE, THBD, and VTN. Gene mutations are screened for through serial single-gene testing, multi-gene panels, or comprehensive genomic testing, employing PCR or chromosomal microarray to perform sequence analysis or copy number analysis [8].

Treatment consists of blood transfusions, electrolyte correction, and nephrotoxic drug discontinuation. Patients may require dialysis for symptomatic uremia. Complement-mediated HUS, in particular, is treated with Eculizumab, a monoclonal antibody to C5 complement, alongside plasma exchange and immunosuppression [4,7]. Ravulizumab, a long-acting variant, is also available for treatment [9]. Outcomes vary considerably, from complete remission of the disease to chronic relapse and kidney failure. Furthermore, individuals with atypical HUS are more likely to develop chronic complications, including persistent hypertension and chronic renal disease [10]. These patients may need lifelong dialysis and kidney transplants [11].

Atypical HUS may be difficult to diagnose and properly identify in a timely manner due to its overlap with other TMA syndromes. A timely diagnosis is necessary to initiate the correct treatment, thereby directly affecting clinical outcomes. The diagnosis of these conditions is also hindered by the lack of specific tests available to accurately diagnose each condition, thereby imposing limitations in clinical practice. The following case highlights these difficulties and provides insight into areas of improvement.

## Case presentation

A 61-year-old female with a history of metastatic peritoneal papillary serous carcinoma of the ovary presented to Holy Cross Hospital on 12/18/19 due to symptomatic anemia. She was initially diagnosed with stage IV peritoneal carcinoma in June of 2015. She was treated over the past several years by a combination of surgical debulking, multiple different chemotherapeutic regimens, and symptomatic management with paracentesis. She was most recently treated on a regimen of gemcitabine and paclitaxel, having received her last treatment on 12/11/19. During post-chemotherapeutic follow-up on 12/18/19, the patient complained of worsening shortness of breath and fatigue. She was found to have a decreased hemoglobin (Hgb) and platelet count. She received two units of packed red blood cells and was transferred to the emergency department of Holy Cross Hospital. 

On presentation to the emergency department, the patient had a blood pressure of 229/94 mmHg. Physical exam demonstrated mild tenderness in the left upper quadrant and alopecia. Abnormal lab values were as shown in Table 1. 

**Table 1:** Lab values on 12/18/19

Lab	Value	Reference Range
Hemoglobin	6.3 g/dL	11.9-14.8 g/dL
Hematocrit	17.30%	35-43%
Platelets	41 x 10^9/L	153-361 x 10^9/L
Sodium	129 mEq/L	136-145 mEq/L
Blood Urea Nitrogen	45 mg/dL	8-20 mg/dL
Creatinine	3.14 mg/dL	0.50-1.10 mg/dL
Total Bilirubin	2.0 mg/dL	0.3-1.0 mg/dL

A stool guaiac test was negative. Urinalysis revealed proteinuria and hematuria. Chest X-ray showed cardiac enlargement. A renal ultrasound was normal. A blood smear was positive for anisocytosis and schistocytes. Based on these results, the patient was diagnosed with MAHA. Due to her worsening anemia and acute kidney injury, the patient was admitted for further treatment. She was started on IV hydration with normal saline, and she was also given an additional two units of packed red blood cells. The patient was offered dialysis and refused at this time. Labs drawn on 12/19/19 revealed an elevated lactate dehydrogenase and decreased haptoglobin. She was given IV steroids without improvement. She was subsequently started on daily plasmapheresis on 12/21/19. Tests for complement and ADAMTS13 were drawn. The patient received her last round of plasmapheresis on 12/26/19 and was stable for discharge on 12/27/19. The patient’s complement levels showed a decrease in C3, C4, and CH50. ADAMTS13 activity was found to be normal. She had follow-up labs drawn on 12/31/19. The abdominal ultrasound on 12/31/19 showed heterogeneous hepatic echotexture and a small amount of abdominal free fluid with no other acute findings. A blood smear showed fragmented red blood cells. Lab results returned on 1/2/20 and are shown in Table 2. 

**Table 2:** Lab values on 1/2/20

Lab	Value	Reference Range
Hemoglobin	8 g/dL	11.9-14.8 g/dL
White blood cell Count	15 x 10^9/L	3.8-10.4 x 10^9/L
Platelets	103 x 10^9/L	153-361 x 10^9/L
Blood Urea Nitrogen	80 mg/dL	8-20 mg/dL
Creatinine	5.53 mg/dL	0.50-1.10 mg/dL
eGFR	8 mL/min	>60 mL/min
Total bilirubin	1.5 mg/dL	0.3-1.0 mg/dL
Lactate dehydrogenase	894 U/L	80-225 U/L
C3	Normal	Normal
C4	Low	Normal
CH50	Normal	Normal

Due to lab derangements, the patient was asked to return to the office. On office presentation, 1/2/20, she was noted to have pallor and anasarca thought to be due to fluid overload secondary to worsening kidney function. Repeat Hgb and platelets were once again low, and she was readmitted for further treatment. Lab testing for ADAMTS13 was negative, and thrombotic thrombocytopenic purpura was ruled out. Complement values were decreased, and the patient’s diagnosis was revised to atypical HUS. Genetic testing revealed a mutation in the thrombomodulin D59.3 gene, confirming the diagnosis. She was started on treatment with eculizumab and hemodialysis. She was administered the meningococcal vaccine due to eculizumab-induced C5 inhibition. Throughout her hospital stay, she made significant improvements and was able to be discharged on 1/10/20. Her most recent lab values before discharge are shown in Table 3. 

**Table 3:** Lab values on 1/10/20

Lab	Value	Reference Range
Hemoglobin	8.7 g/dL	11.9-14.8 g/dL
Platelets	138 x 10^9/L	153-361 x 10^9/L
Creatinine	3.9 mg/dL	0.50-1.10 mg/dL
Blood urea nitrogen	18 mg/dL	8-20 mg/dL
eGFR	12 mL/min	>60 mL/min

At the time of writing, this patient is currently doing well. Her most recent lab values are shown in Table 4. 

**Table 4:** Lab values on 11/23/21

Lab	Value	Reference Range
Hemoglobin	11.9 g/dL	11.9-14.8 g/dL
Hematocrit	34.70%	35-43%
Platelets	266 x 10^9/L	153-361 x 10^9/L
Sodium	139 mEq/L	136-145 mEq/L
Potassium	4.3 mEq/L	3.5-5.0 mEq/L
Chloride	103 mEq/L	98-106 mEq/L
Blood Urea Nitrogen	23 mg/dL	8-20 mg/dL
Creatinine	1.57 mg/dL	0.50-1.10 mg/dL
eGFR	35 mL/min	>60 mL/min
Total Bilirubin	0.6 mg/dL	0.3-1.0 mg/dL

After multiple doses of eculizumab, the patient regained sufficient renal function to discontinue hemodialysis treatment. On her most recent follow-up, she has been in stable condition and has begun the process of tapering off from eculizumab to long-term conservative management.

## Discussion

The patient, in this case, was determined to have atypical HUS due to a mutation of the thrombomodulin D59.3 gene. This mutation combined with overactivation of the coagulation cascade by complement increases the risk for microthrombi formation. This leads to severe anemia, thrombocytopenia, and renal failure. According to the International Society of Nephrology, a complement abnormality is found in at least 50% of patients with atypical HUS, resulting in complement overactivation through alternative pathway dysfunction [2].

Complement activation is typically a crucial part of innate immunity and can be activated via three distinct pathways: classical, lectin, and alternative. All three pathways lead to the production of anaphylatoxins, opsonization of pathogens, and formation of the membrane attack complex (MAC). Among these three pathways, only the alternative pathway can be activated spontaneously, which means it needs to be tightly regulated. Mutations that cause overactivation can lead to excessive MAC formation. MAC, in turn, activates endothelial cells and pro-coagulative tissue factors. This initiates the coagulation cascade, creating microthrombi within capillaries and arterioles [1].

Several case reports describe the presentation of atypical HUS in direct response to chemotherapeutic regimens such as sunitinib and gemcitabine [12-13]. However, this patient had received multiple doses of gemcitabine over several months before this without these complications. Additionally, drug-induced thrombotic microangiopathy, a separate subset of TMA, may be considered in such a case, as the non-immune variant may present weeks to months after drug initiation with similar symptoms of fatigue and renal injury. In this disorder, the theorized mechanism is dose-dependent toxicity which develops over weeks to months. However, in contrast to atypical HUS, evidence of severe complement derangements are typically absent [14]. As there is limited evidence on the complete pathophysiology of this condition, and considering the significant overlap with other TMAs, it may be that these two conditions are not entirely separate entities. Further research is needed to help understand the distinctions between the two diseases [15].

Cancer-induced HUS has also been observed in several cases. These cases have demonstrated a positive correlation between the progression of cancer and increasing hemolytic disease [16]. However, as this patient’s cancer diagnosis remained relatively stable, this positive correlation was not present. Nonetheless, this cancer diagnosis also posed a limitation in the timely diagnosis of the patient’s condition as it complicated the patient's clinical presentation. This was further compounded by the patient’s initial refusal of dialysis during her initial hospitalization.

During her hospitalization, this patient’s worsening anemia was complicated by her cancer diagnosis and her chemotherapeutic regimen, causing delays in diagnosis and treatment. Without prompt intervention, patients can progress to kidney failure, require long-term dialysis, and possibly need a kidney transplant. Additionally, the long-term sequelae of uncontrolled anemia may lead to significant consequences, including chronic heart failure and death. Furthermore, the efficacy of intervention with specific treatment such as eculizumab is high, and prompt treatment provides significant improvement in patient outcomes [7]. Therefore, prompt diagnosis can be course-altering.

This case is unique because it demonstrated a rare diagnosis in the presence of multiple confounding factors. This case illustrates that MAHA may need to be approached broadly and then narrowed based on specific test results, which can help to determine a definitive diagnosis. As this case demonstrates, the difficulty in diagnosis of the condition and the multiple specific tests necessary for confirmation may lead to misdiagnosis, especially when testing is limited. For example, previous case reports were limited in genetic testing, which was available in this case. The availability of genetic testing provided a clearer diagnosis of atypical HUS and helped to eliminate alternative diagnoses. Additionally, the etiology of the disease is multifactorial, and the genetic abnormalities that predispose it, superimposed on the environmental factors that may trigger it, are widely variable. As we continue to elucidate common genetic abnormalities associated with the disease and the availability of genetic testing increases, this diagnosis may become more readily available. As genetic testing becomes more readily available, cross-sectional studies may be performed to further evaluate the prevalence and incidence of genes associated with atypical HUS. The environmental triggers of this disease may also be further elucidated through studies on genetically modified animals exposed to potential triggers.

## Conclusions

This case demonstrates a rare diagnosis of atypical HUS, which was incompletely diagnosed initially as MAHA and thrombocytopenia because the patient was complicated, and the detailed testing required to confirm a case of atypical HUS was very specific and less readily available. On a broader note, complicated patients present a challenge for clinical diagnosis as they have multiple confounding factors that suggest a different diagnosis. This case demonstrates that atypical HUS must always be included in the differential diagnosis of an adult with suspected MAHA. All resources and testing available to clinicians must be considered, as these tools help to definitively confirm the diagnosis and provide insight into further management of patients. This improves outcomes and reduces morbidity and mortality.
